# Non-COVID-19 hospitalization and mortality during the COVID-19 pandemic in Iran: a longitudinal assessment of 41 million people in 2019–2022

**DOI:** 10.1186/s12889-024-17819-0

**Published:** 2024-02-05

**Authors:** Mahya Razimoghadam, Mehdi Yaseri, Mehdi Rezaee, Aliakbar Fazaeli, Rajabali Daroudi

**Affiliations:** 1 Department of Health Management, Policy and Economics, School of Public Health, Tehran University of Medical Sciences, Tehran, Iran; 2 Department of Epidemiology and Biostatistics, School of Public Health, Tehran University of Medical Sciences, Tehran, Iran; 3 Department of Orthopedics, School of Medicine, Tehran University of Medical Sciences, Tehran, Iran; 4 National Center for Health Insurance Research, Tehran, Iran

**Keywords:** COVID-19, Non-COVID-19, Hospitalization, Mortality, Iran health insurance organization, Injuries, Respiratory diseases, Mental health, Cardiovascular disease

## Abstract

**Background:**

During a COVID-19 pandemic, it is imperative to investigate the outcomes of all non-COVID-19 diseases. This study determines hospital admissions and mortality rates related to non-COVID-19 diseases during the COVID-19 pandemic among 41 million Iranians.

**Method:**

This nationwide retrospective study used data from the Iran Health Insurance Organization. From September 23, 2019, to Feb 19, 2022, there were four study periods: pre-pandemic (Sept 23-Feb 19, 2020), first peak (Mar 20-Apr 19, 2020), first year (Feb 20, 2020-Feb 18, 2021), and the second year (Feb 19, 2021-Feb 19, 2022) following the pandemic. Cause-specific hospital admission and in-hospital mortality are the main outcomes analyzed based on age and sex. Negative binomial regression was used to estimate the monthly adjusted Incidence Rate Ratio (IRR) to compare hospital admission rates in aggregated data. A logistic regression was used to estimate the monthly adjusted in-hospital mortality Odds Ratio (OR) for different pandemic periods.

**Results:**

During the study there were 6,522,114 non-COVID-19 hospital admissions and 139,679 deaths. Prior to the COVID-19 outbreak, the standardized hospital admission rate per million person-month was 7115.19, which decreased to 2856.35 during the first peak (IRR 0.40, [0.25–0.64]). In-hospital mortality also increased from 20.20 to 31.99 (OR 2.05, [1.97–2.13]). All age and sex groups had decreased admission rates, except for females at productive ages.

Two years after the COVID-19 outbreak, the non-COVID-19 hospital admission rate (IRR 1.25, [1.13–1.40]) and mortality rate (OR 1.05, [1.04–1.07]) increased compared to the rates before the pandemic. The respiratory disease admission rate decreased in the first (IRR 0.23, [0.17–0.31]) and second years (IRR 0.35, [0.26–0.47] compared to the rate before the pandemic. There was a significant reduction in hospitalizations for pneumonia (IRR 0.30, [0.21–0.42]), influenza (IRR 0.04, [0.03–0.06]) and COPD (IRR 0.39, [0.23–0.65]) during the second year. There was a significant and continuous rise in the hematological admission rate during the study, reaching 186.99 per million person-month in the second year, reflecting an IRR of 2.84 [2.42–3.33] compared to the pre-pandemic period.

The mortality rates of mental disorders (OR 2.15, [1.65–2.78]) and musculoskeletal (OR 1.48, [1.20–1.82), nervous system (OR 1.42, [1.26–1.60]), metabolic (OR 1.99, [1.80–2.19]) and circulatory diseases (OR 1.35, [1.31–1.39]) increased in the second year compare to pre-pandemic. Myocardial infarction (OR 1.33, [1.19–1.49]), heart failure (OR 1.59, [1.35–1.87]) and stroke (OR 1.35, [1.24–1.47]) showed an increase in mortality rates without changes in hospitalization.

**Conclusions:**

In the era of COVID-19, the changes seem to have had a long-term effect on non-COVID-19 diseases. Countries should prepare for similar crises in the future to ensure medical services are not suspended.

**Supplementary Information:**

The online version contains supplementary material available at 10.1186/s12889-024-17819-0.

## Introduction

The World Health Organization (WHO) declared the outbreak of the new Coronavirus as a public health emergency on January 30, 2020. Later, it was announced on March 11, 2020, that COVID-19 had become a pandemic [1]. Iranian officials confirmed the first positive SARS-CoV-2 infection on February 19, 2020. SARS-CoV-2 virus has infected over 7.6 million Iranians, with over 146,480 deaths reported since Aug 2023 [2].

As the pandemic spread and global efforts were made to prevent it, the supply and demand of health care were affected. As a result of cancelling elective activities in hospitals and diverting resources toward COVID-19 infected patients, as well as the patients’ fear of visiting hospitals and their decisions to delay receipt of medical care, the number and pattern of hospital visits changed. Indirect effects, or the collateral effects of the COVID-19 pandemic, refer to the changes that have occurred in the provision of health services and consumption because of the pandemic [3–5].

Indirect effects vary depending on patient characteristics, type of services, and pandemic management in each country [3]. Indirect effects are important because they affect non-COVID-19 patients’ morbidity and mortality indirectly because of changes in health care delivery, patient behaviors, and lifestyle choices during pandemics [3, 6, 7]. This study investigates hospital admissions and mortality in non-COVID-19 diseases during the 2 years after the COVID-19 outbreak in Iran, to reveal the short- and long-term effects of this pandemic on other diseases.

## Method

### Data source

This retrospective observational study was conducted using Iranian Health Insurance Organization (IHIO) hospital claims data. IHIO covers half of Iran’s population, 41,548,604 million people, making it one of Iran’s two major primary insurances. This insurance is inclusive since it has five different funds for various populations. A wide range of groups are served by these funds, including public employees, those in rural areas and sparsely populated cities, as well as students, the homeless, and the impoverished. IHIO has contracts with 956 hospitals across all provinces of Iran, which represents more than 88% of Iran’s hospitals.

A nationwide mandate requires all hospitals in Iran to transmit electronic medical records to the Iran electronic health record system (SEPAS). This is administered by the Ministry of Health and Medical Education (MOHME) of Iran. SEPAS serves as a national database that gathers electronic medical records from all health-care facilities using standardized data exchange protocols. MOHME’s information technology and statistics management center has developed these protocols. Therefore, SEPAS bridges hospitals and insurance companies. An electronic system called Rose is used for processing inpatient records in the IHIO. Since 2018 Rose has been established to receive medical records and billing data from contracted hospitals through SEPAS. MOHME’s information technology and statistics management center and the department of statistics, information technology, and communication within the IHIO standardize medical records and assure data quality. The National Center for Health Insurance Research (NCHIR) in IHIO, maintains access to the IHIO database for researchers. To conduct current research, NCHIR facilitates the extraction of claims data from the Rose database. The claims data include patients’ characteristics, diagnosis codes, and hospital spending and outcome details.

Data were extracted from Rose database. At the beginning of the study, 7,977,193 hospital inpatient records were retrieved. For the purpose of analysis, the data have been cleaned and categorized. Data missing included 878,787 records without diagnosis codes, which were excluded from the study. Across time periods, data are missing randomly. Furthermore, the patients’ characteristics in the excluded records did not differ significantly from those in the included records. There are two types of hospitalization in Iran. The first consists of normal hospitalizations that last at least one night, and the second includes short-term hospitalizations lasting less than 6 hours. This study examined records of both types of hospitalization. Outpatient admissions and emergency admissions resulting in no hospitalization were excluded from the study. In the supplementary material, fig. S1 contains the study population flowchart.

### Study periods and COVID-19 events

The study period ranged from September 23, 2019, to February 19, 2022. A detailed description of 29 months of study was provided in the descriptive section of the study. For comparison analysis, four different time periods were named and selected for deep investigation: The first period encompassed the period before the COVID-19 outbreak and ran from September 23, 2019, to February 19, 2020. The second period was between March 20 and April 19, 2020, when COVID-19 hospitalizations in Iran peaked for the first time. This period was of special significance, both regarding the initial peak of COVID-19 cases in Iran and the full implementation of nationwide lockdowns. We extracted this time because it represents the pure effects of lockdowns. This was the only period during which Iran implemented COVID-19 lockdowns nationwide, including the cancellation of in-hospital elective surgeries.

**Fig. 1:**
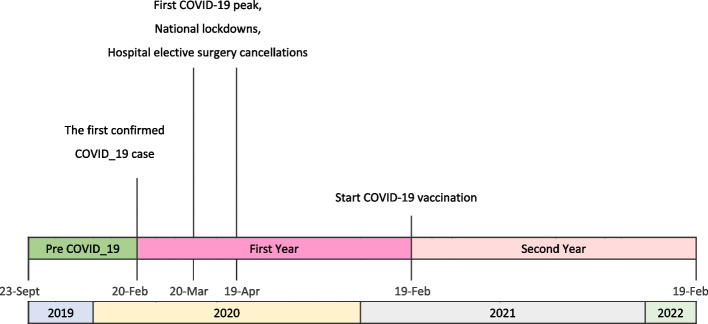
Timeline of the Study and COVID-19 Pandemic Events in Iran

This study also determined two other time periods. The third period was the first year following the COVID-19 outbreak in Iran, from February 20, 2020, to February 18, 2021. From the time the first SARS-CoV-2 case in Iran was confirmed by the government, this time period began. The third period explains the whole first year of the pandemic including all COVID-19 waves and restrictions policies. Therefore, during this first year of the pandemic, there were also the first peak period. The fourth period was defined as the second year after the outbreak. It began on February 19, 2021, and ended on February 19, 2022. Because the COVID-19 vaccine was first injected in Iran in February 2021, the second year could also be considered the post-vaccination year. We have considered mass vaccination as a way to normalize hospital activities and modify community behaviors. This includes reducing fear and delaying essential health care [8]. Figure 1 provides information about the study timeline and relevant events.

### Variables and outcomes

In this study, the outcomes are the number of hospital admissions, the admission rate, and the incidence rate ratio (IRR) for comparison purposes. The other outcomes are the number of in-hospital deaths, in-hospital mortality rate, and odds ratio (OR) for comparison. The information regarding patient in-hospital mortality was extracted from the discharge status section of the hospital records. It is considered in-hospital mortality if the patient’s discharge status indicates “dead”.

Hospital admissions and mortality were stratified by age, sex, and disease. Patients were divided into five age groups: under 5 years old, 5–14, 15–44, 45–64, and over 64. Sex was classified into male and female based on birth determination. The 21 disease groups were based on the primary diagnosis codes in the patients’ records. These codes were categorized according to chapters of the International Classification of Diseases 10th Revision (ICD 10th). In addition, six selected diseases were investigated separately including Pneumonia, Influenza, Chronic Obstructive Pulmonary Disease (COPD), Myocardial infarction, Heart failure and Stroke. The primary diagnosis codes used in this study identify the most severe and/or resource-intensive diagnosis during hospitalization or an inpatient encounter. To ensure the focus on non-COVID-19 conditions, patients with U07.1 and U07.2 as their primary diagnosis codes, indicating COVID-19, were excluded from the study. Only in the second figure of this study, we use excluded data including COVID-19 hospital admissions to figure out the peaks and compare. It’s imperative to note that while this study only considered primary diagnosis codes, patients may have other underlying health conditions not specifically addressed here. Consequently, non-COVID-19 patients might have COVID-19 listed as a secondary diagnosis. The ICD 10th codes used in the study are presented in Table S1 of the supplement.

## Statistical method

The overall standardized admission rate was calculated by dividing the number of monthly admissions by the number of insurance members of the same age and sex. Direct standardization was performed based on the age and sex distribution of IHIO members in each time period. Hospital admission rate is reported at millions of IHIO members per month. After aggregating the individual data, the adjusted IRRs were calculated using negative binomial regression. The age and sex distribution of IHIO members used as exposure variables to estimate the adjusted IRR. The baseline period was measured in two ways. In the first instance, we considered the period before the COVID-19 outbreak as the baseline. In this case, the first peak, the first year, and the second year were each compared to the period before the pandemic. In the second instance, the first year of the pandemic was regarded as the baseline. In this case, the admission rate in the second year of the pandemic was compared with the first year of the pandemic.

In-hospital mortality rates were calculated by dividing the number of monthly deaths by the total number of admissions in the same age, sex, and disease category. This rate is presented in units of 1000 admissions per month. A logistic regression model was used to estimate the adjusted OR to compare mortality rates at different times. Age and sex variables are entered into each regression analysis for estimating adjusted ORs. The OR calculations were also based on two baseline periods, just like the IRR calculations. Stata Statistical Software Release 17 College Station, TX: StataCorp LLC was used for statistical analysis. All analyses were conducted with a 95% confidence level. The statistical significance level was set at p 0.05. The Benjamini-Hochberg procedure was applied to reduce false discovery rates and create adjusted *p*-values.

## Results

A total of 6,522,114 non-COVID-19 admissions to hospitals were made by 4,260,266 patients during the 29 months of the study. Prior to the COVID-19 outbreak, there were 200,008 monthly admissions, and during the first peak, 99,588 admissions were recorded. The monthly average of admissions in the first year of the pandemic was 184,779, and in the second year it was 275,394. Table S2 and S3 provide further details regarding the number of monthly admissions by age, sex, and disease in the supplement.

In total, 139,679 non-COVID-19 deaths were recorded. There were 4041 hospital deaths per month before the pandemic; in the first peak of COVID-19, 3186 deaths occurred. Monthly deaths were recorded at 4274 in the first year, and at 5683 in the second year. In the supplement, tables S4 and S5 provide details about the number of monthly in-hospital deaths by age, sex, and disease.

Prior to the COVID-19 outbreak, the standardized hospital admission rate for non-COVID-19 diseases per a million person-month was 7115.19, which reached 2856.35 during the first peak. The first-year admission rate was 5731.53, and the second-year admission rate was 8891.36. In the supplement, tables S6 and S7 show hospital admission rates by age, sex, and disease.

The in-hospital mortality rate for non-COVID-19 diseases per 1000 admission-month was 20.20 before the pandemic and 31.99 during the first peak. Mortality rates in the first and second year of the pandemic averaged 23.13 and 20.63, respectively. Tables S8 and S9 in the supplement provide mortality rates by age, sex, and disease for each period.

**Fig. 2:**
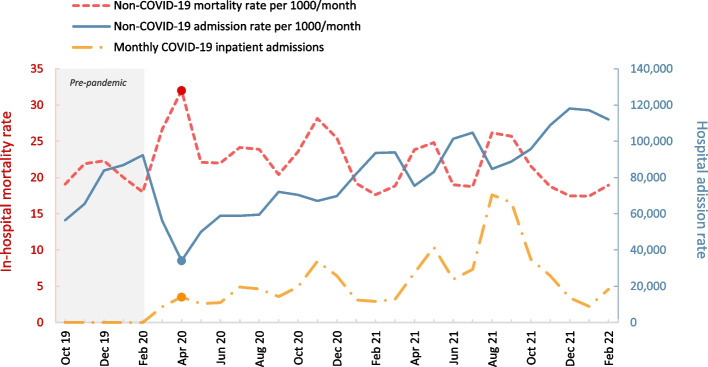
Standardized non-COVID-19 hospital admission rate per 1000 person-month, mortality rate per 1000 admissions-month and absolute number of monthly COVID-19 inpatient admissions from Sept 23, 2019 to Feb 19 2022. A filled marker indicates the first peak of COVID-19 between 20 Mar and 19 Apr 2020

**Fig. 3:**
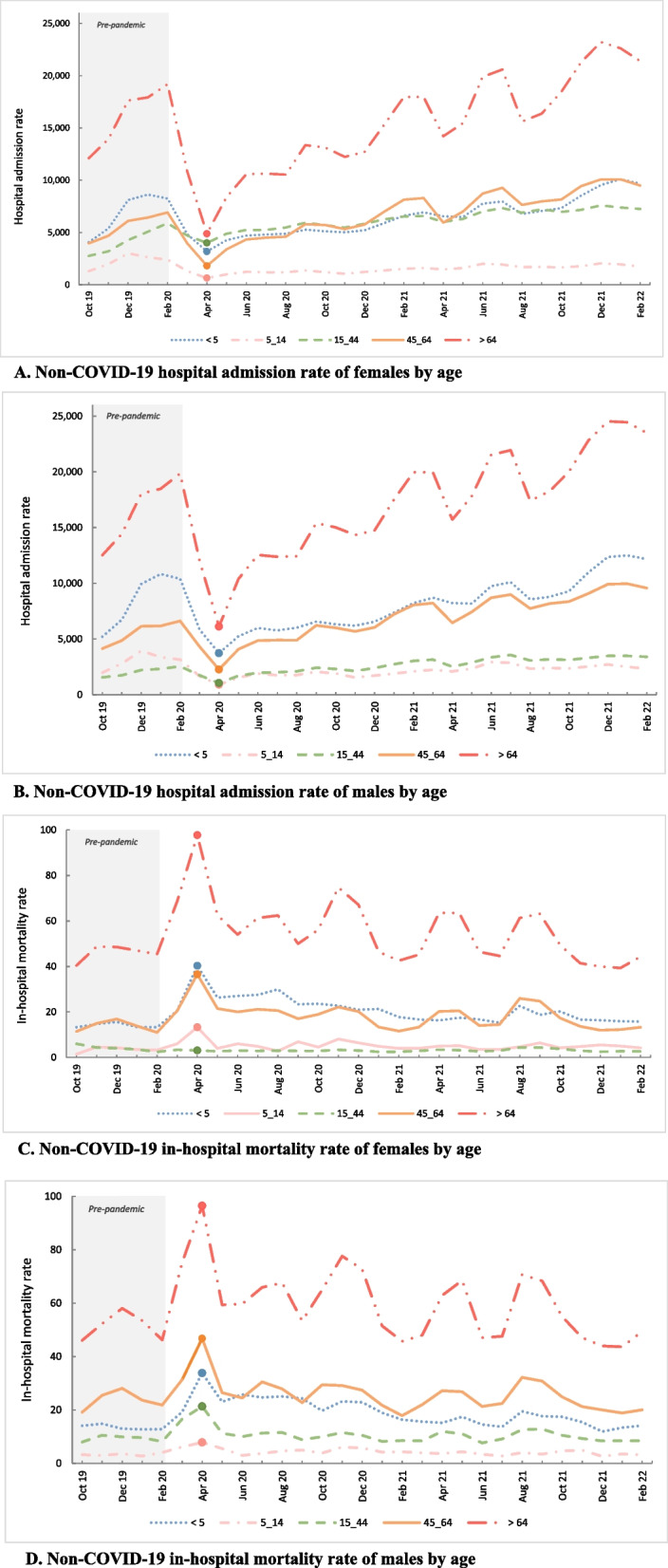
Non-COVID-19 hospital admission rate per million/month (**A**, **B**) and non-COVID-19 in-hospital mortality rate per 1000/month (**C**, **D**) by age and sex from Sept 23, 2019 to Feb 192,022. A filled marker indicates the first peak of COVID-19 between 20 Mar and 19 Apr 2020

Figure 2 illustrates the non-COVID-19 hospital admission rate and associated in-hospital mortality rates, and the COVID-19 monthly hospital admissions. Figure 3 shows the non-COVID-19 hospital admission rate and in-hospital mortality rate, categorized by age and sex of patients.

### The first peak of COVID-19 compared to the pre-pandemic period

**Fig. 4:**
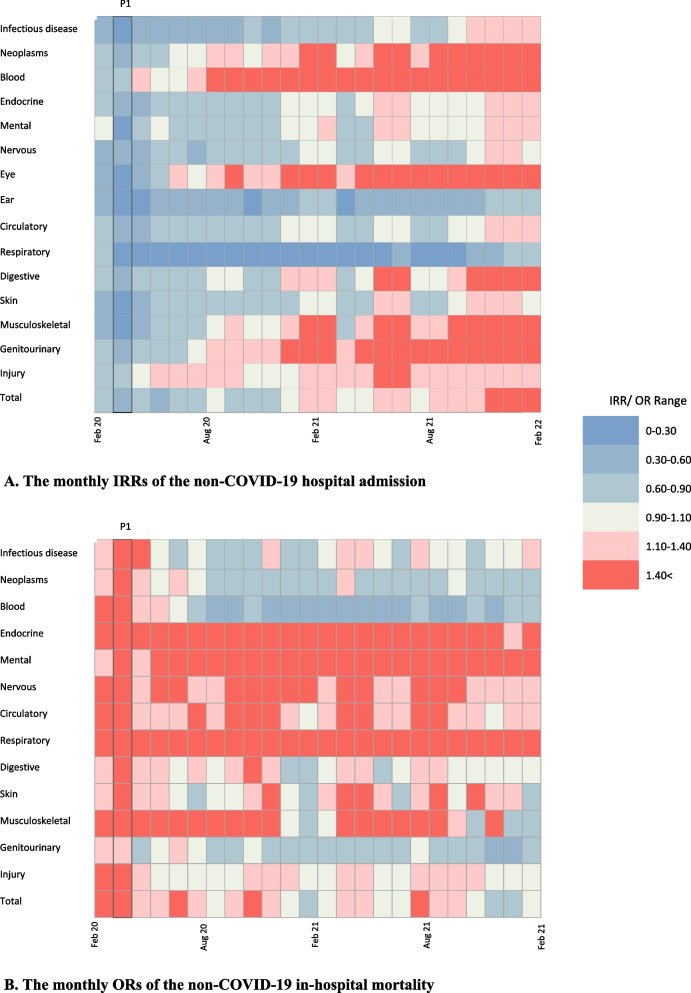
The monthly IRRs (**A**) of hospital admission and ORs (**B**) of in-hospital mortality in non-COVID-19 conditions after the COVID-19 outbreak, from Feb 20, 2020, to Feb 19, 2022, compared to the pre-pandemic period (P1 indicates the first peak of COVID-19 between 20 Mar and 19 Apr 2020)

The non-COVID-19 hospital admission rate decreased during the first COVID-19 peak in Iran (IRR 0.40, [0.25–0.64]). The largest reductions were found in eye (IRR 0.04 [0.02–0.11]) and ear diseases (IRR 0.09 [0.05–0.16]). When compared to the rates before the pandemic, the admission rates for infectious (IRR 0.21 [0.11–0.40]), respiratory (IRR 0.15 [0.08–0.28]), musculoskeletal (IRR 0.14 [0.08–0.26]), metabolic (IRR 0.32, [0.16–0.62]), mental (IRR 0.30, [0.12–0.72]), nervous system (IRR 0.30, [0.19–0.47]), circulatory (IRR 0.33, [0.11–0.98]), digestive (IRR 0.32, [0.20–0.54]), skin (IRR 0.23, [0.15–0.35]), and genitourinary diseases (IRR 0.47, [0.24–0.89]), as well as injuries and poisonings (IRR 0.62, [0.44–0.89]), were reduced during the first peak of COVID-19. Moreover, admission rates for pneumonia (IRR 0.15 [0.07–0.30]), COPD (IRR 0.16 [0.06–0.46]), and influenza (IRR 0.05 [0.02–0.10]) decreased. Figure 4A shows the IRRs of non-COVID-19 diseases in each month of the study compared to the time before the pandemic.

During the first peak of COVID-19 in Iran, in-hospital mortality rates for other diseases increased (OR 2.05, [1.97–2.13]). Mortality rates of infectious (OR 1.91, [1.58–2.30]), neoplasms (OR 1.45, [1.22–1.72]), metabolic (OR 3.10, [2.47–3.88]), circulatory (OR 2.62, [2.43–2.82]), respiratory (OR 3.28, [2.93–3.68]), digestive (OR 1.96, [1.62–2.35]), and urogenital diseases (OR 1.39, [1.16–1.68]), as well as injury and poisonings (OR 1.73 [1.49–2.02]), increased in the first peak compared to the pre-pandemic period. A rise in the non-COVID-19 mortality rate in the first peak was also observed in pneumonia (OR 3.36, [2.83–4.00]), myocardial infarction (OR 1.31, [1.00–1.71]) and stroke (OR 1.59, [1.29–1.96]). In most periods, deaths from eye and ear diseases were zero, so they were excluded from the OR analysis. Figure 4B shows the ORs of non-COVID-19 diseases in each month of the study compared to the time before the pandemic. Details of IRRs and ORs at the first peak compared with pre-pandemic levels are presented in tables S10–S13 of the supplement.

### The first year after COVID-19 outbreak compared to the pre-pandemic period

Compared to the pre-pandemic period, non-COVID-19 hospital admission rates decreased in the first year after the outbreak (IRR 0.80, [0.73–0.89]). The most significant decreases were in respiratory (IRR 0.23, [0.17–0.31]), infectious (IRR 0.55, [0.40–0.76]), ear (IRR 0.36, [0.28–0.46]), nervous system (IRR 0.66, [0.53–0.81]) and skin diseases (IRR 0.69, [0.56–0.84]). Hospital admission rates for influenza (IRR 0.06, [0.04–0.10]), pneumonia (IRR 0.18, [0.13–0.26]) and COPD (IRR 0.27 [0.16–0.46]) declined compared to pre-pandemic levels. However, the admission rate for blood diseases increased in the first year following the pandemic (IRR 1.50, [1.24–1.83]).

Over the first year of the pandemic in Iran, non-COVID-19 diseases recorded an increase in mortality rate (OR 1.26, [1.23–1.28]). Compared to the pre-pandemic era, in-hospital mortality rates increased in metabolic (OR 2.03, [1.83–2.24]), circulatory (OR 1.42, [1.38–1.47]), respiratory (OR 2.81, [2.68–2.95]) and digestive diseases (OR 1.16, [1.08–1.24]), along with injuries and poisonings (OR 1.18 [1.10–1.27]). Conversely, the mortality rate of neoplasms (OR 0.93, [0.87–0.99]), and genitourinary diseases (OR 0.89, [0.82–0.96]) decreased.

Pneumonia (OR 3.10, [2.86–3.36]), COPD (OR 1.94, [1.71–2.20]), influenza (OR 1.85, [1.41–2.43]), myocardial infarction (OR 1.16, [1.03–1.30]), heart failure (OR 1.55, [1.31–1.84]) and stroke (OR 1.22, [1.12–1.34]) had higher mortality rates in the first year than before the pandemic. Tables S14–S17 of the supplement show IRRs and ORs based on age, sex, and disease for the first year compared to the pre-pandemic period.

### The second year after the COVID-19 outbreak compared to the pre-pandemic period

In the second year after the COVID-19 outbreak in Iran following vaccination, the hospital admission rate increased compared to the time before the pandemic (IRR 1.25, [1.13–1.40]). Hospital admission rates for neoplasms (IRR 1.52, [1.09–2.13]), blood (IRR 2.84, [2.42–3.33]), eye (IRR 2.63, [1.62–4.27]), digestive (IRR 1.30, [1.04–1.64]), musculoskeletal (IRR 1.60, [1.17–2.20]), and genitourinary diseases (IRR 1.79, [1.30–2.49]), increased along with injuries (IRR 1.39, [1.20–1.60]).

A reduction in admission rates was still evident in the second year for both ear (IRR 0.52, [0.41–0.66]) and respiratory diseases (IRR 0.35, [0.26–0.47]). Similarly, influenza (IRR 0.04, [0.03–0.06]), pneumonia (IRR 0.30, [0.21–0.42]) and COPD (IRR 0.39, [0.23–0.65]) admissions were lower than prior to the pandemic. In the 2 years following the COVID-19 outbreak, hospital mortality rates for some diseases were still increasing. The mortality rate for non-COVID-19 diseases increased in the second year compared to the time before the pandemic (OR 1.05, [1.04–1.07]). Tables S18–S21 in the supplement show details of the IRRs and ORs in the second year compared to the pre-pandemic period.

### The year after vaccination in comparison with the year before vaccination

**Fig. 5:**
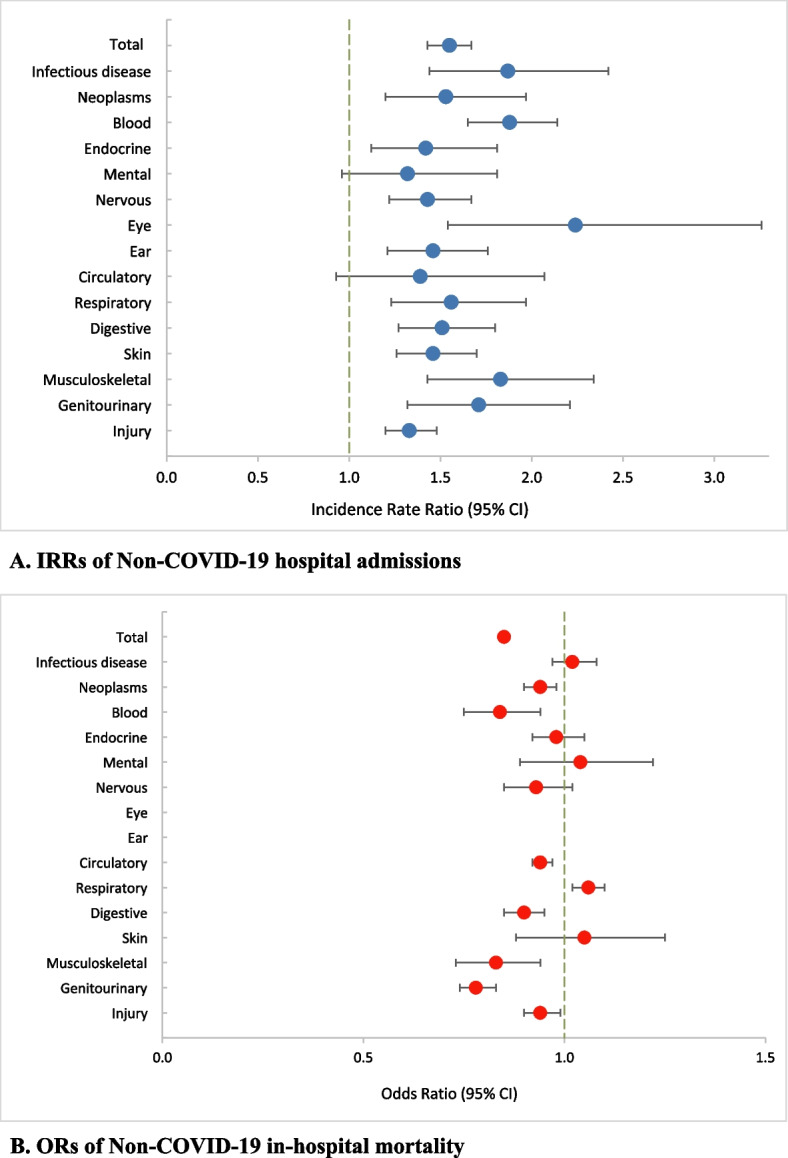
Hospital admissions IRRs (**A**) and in-hospital mortality ORs (**B**) of non-COVID-19 conditions in the second year of the pandemic (Feb 19, 2021 – Feb 19, 2022) compared to the first year (Feb 20, 2020 - Feb 18, 2021) as the baseline

In the second year following COVID-19 vaccination, the non-COVID-19 hospital admission rate (IRR 1.55, [1.43–1.67]) increased and the mortality rate (OR 0.85, [0.84–0.86]) decreased compared to the first year. Figure 5 show the IRRs and ORs for the COVID-19 vaccination year compared to the previous year. Supplementary tables S22–S25 provide more information about changes in hospital admission and in-hospital mortality rates in the second year of the pandemic compared to the first year.

## Discussion

In this study, we attempted to uncover the indirect effects of the COVID-19 outbreak on other diseases using data from 41 million Iranian residents. Iran was one of the first countries affected by COVID-19. Immediately after officially detecting the first disease case on 19 February, 2020, the National COVID-19 Committee in Iran was established. The committee suspended commercial flights from China, issued health certificates to foreign travelers, closed schools and universities, banned public gatherings, and restricted religious gatherings [9, 10]. A 15-day shutdown of all non-essential businesses and services was announced in several provinces on March 19 [11]. On March 20, the Minister of Interior Affairs was directed to closed all malls and markets until April 3 [11]. To control the spread of disease, strict health restrictions were announced on March 25. At the end of March, Iran imposed a travel ban, which prohibited national travels. Residents of a city were identified with proof, such as a national code, a car number, and car insurance [12].

These changes had a significant impact on the health-care system in many ways, particularly in relation to healthcare demand and supply. During the first COVID-19 peak from March 20 to April 19, 2020, the non-COVID-19 admission rate decreased by 59.9%. Health-care services were disrupted because of the non-provision of elective services, the absence or insufficient time of medical staff, the lack of enough beds and medical equipment, and the closure of outpatient centers and doctors’ offices. In most countries, according to WHO reports, supply-side factors had a greater effect on services than demand-side factors, especially in middle- and low-income countries [7, 13].

During COVID-19’s pandemic, the Iranian health system was greatly affected. This was due to a shortage of sufficient Personal Protective Equipment (PPE), ICU beds, available trained medical staff, diagnostic tests, and pharmaceuticals at the onset of the pandemic [10]. For instance, a range of 5 to 10% of nurses have been allocated from various hospital wards to handle COVID-19 cases [14]. Moreover, there was no proper protocol for screening, temporary health centers, and rehabilitation of COVID-19 patients [15]. Consequently, patients and health staff were confused in hospitals even in cases unrelated to COVID-19.

The demand for services also decreased, mainly due to fear of medical centers, especially hospitals. Additionally, because out-of-pocket payments are high in Iran, and the COVID-19 pandemic adversely affected households’ incomes and employment rates, some patients may have declined hospital treatments. Iran experienced severe economic difficulties as a consequence of the coincidence of the COVID-19 pandemic and US sanctions [16]. In addition, exports were disrupted because some countries closed their borders to Iran, putting economic pressure on many small and medium-sized businesses [12, 16]. Iran also failed to provide financial assistance to these businesses during the quarantine [12]. Moreover, travel restrictions prevented many patients in rural and small cities from receiving proper medical care. Because the inequity of health sector resources within Iran’s provinces makes it necessary to travel to a developed region to receive health care [17–22].

There is often discussion regarding the consequences of delaying elective services. It is unclear which actions directly affected people’s health and which actions were unnecessary. Iran’s hospitals closed many eyes and orthopedic surgeries during the first peak of COVID-19. The cancellation of these elective practices may have put people’s health at risk or may have just reduced overtreatment and overdiagnosis [23].

There was a significant decrease in non-COVID-19 hospital admission rates during the first peak of COVID-19 in all age and sex groups, except for females of reproductive age. During the first peak, pregnancy admissions did not decrease significantly either. This may be attributed to the necessity of this group’s hospital visits or to the Iranian government’s efforts to encourage childbearing. Several studies around the world also reported fewer total non-COVID hospital admissions during the first wave [8, 24–40]. This study also demonstrated a significant decline, especially in pediatric hospital admissions during the pandemic, particularly during the first peak. The decrease could be explained by lockdowns and social distancing, which resulted in the closure of schools and preschools. In addition, the implementation of better hygiene practices and the use of face masks have reduced infectious diseases spread among children. Another possible explanation is the fear of parents admitting their vulnerable children to hospitals during the COVID-19 pandemic. Furthermore, the advantages arising from the pandemic, such as improved air quality and increased parental supervision, may have contributed to protecting children from diseases and adverse health conditions. A number of studies around the world have discussed the decline in hospital admissions among children [41–49].

Non-COVID-19 mortality increased by 58.4% after admissions decreased during the first peak of COVID-19 for all age and sex groups, except for women of reproductive age. These findings indicate that patients with severe health conditions attended hospitals, while those in better health postponed admission. The hospital’s shift of resources and equipment to COVID-19 patients was also likely to result in a reduction in quality of service for non-COVID patients, leading to mortality. An increase in non-COVID-19 hospital mortality rates during COVID-19 peaks and lockdowns has also been reported in other studies [24, 27, 30, 34, 40].

A study of COVID-19’s indirect effects throughout the first year was also conducted. Compared to before the pandemic, admission rates were still lower and mortality rates were higher for non-COVID-19 diseases in the first year after the pandemic. As time has passed, however, some patients resumed their postponed hospital admission and hospitals returned to their regular activities and pandemic management has improved in Iran. Lockdown policies in Iran were less strict after the first COVID-19 wave. Based on the number of COVID-19 patients identified, different areas were colored green, blue, yellow, orange, and red. In each wave, restriction rules were implemented based on this color scheme [50]. At no other time, like the first peak, non-COVID-19 hospital admissions were affected in the first year and declined. The recovery of deferred admissions in the first year is in line with the study in United States [29] and differs from Malta [8].

During the second year of the pandemic, after the COVID-19 vaccine was injected, the non-COVID-19 hospital admission rate increased by 25% compared to pre-pandemic. In this time period, Iran experienced the fifth COVID-19 wave that was really severe due to the delta variant. Although lockdown policies were implemented, non-COVID-19 admission rates did not decline as they did in the first wave [50]. In the second year of the pandemic, hospital rates were higher than in the first year. After receiving the COVID-19 vaccination, people may have felt safe, so they obtained the delayed services [8]. Despite the fact that the COVID-19 vaccination in Iran was done slowly and lasted for 8 months, the second year of the pandemic was severely affected by the vaccination.

It is also important to consider the long-term effects of lifestyle changes during the pandemic era. It has been shown that increasing sedentary lifestyles, stress, and changing nutritional habits during quarantine increase cardiovascular disease and diabetes risk [27, 28]. The mortality rates for all diseases in the first and second years remained higher than before the pandemic. Non-COVID-19 mortality rates in the United States study [32] were also high as the COVID-19 outbreak receded, but they ceased in German y[51].

The decrease in hospital admissions and mortality for respiratory diseases continued during the pandemic. Just before the COVID outbreak, there was an influenza wave that contributed to high respiratory diagnosis outcomes. In many other studies, influenza admissions decreased significantly during the COVID-19 pandemic [29, 51, 52]. Social distance, better hygiene, and mask wearing may have helped control other respiratory diseases. As evidenced by current research and many worldwide studies, the COVID-19 pandemic affects COPD and pneumonia [24, 26–28, 32, 33, 53, 54]. There has been a decrease in COPD and pneumonia admission rates since the COVID-19 outbreak, although pneumonia has become more deadly.

Travel restrictions and business activity restrictions were reduced in the second year of the pandemic, resulting in increased admissions and mortality from injuries. Admissions for neoplasms and blood and digestive diseases increased in the second year of the pandemic. It is alarming how rapidly admissions for blood diseases have increased in Iran, especially because reports link COVID-19 to hematological conditions including autoimmune hemolytic anemia [55].

There was an increase in mental disorders and nervous system, metabolic, and cardiovascular disease mortality in the second year of the pandemic. Specifically, myocardial infarction, heart failure, and stroke, without changes in the number of referrals, showed an increase in hospital mortality rates, contrary to studies in Switzerland [56] and England [57]. Several studies have shown that cardiovascular disease mortality increased after the COVID-19 pandemic although the extent of this increase remains unclear [24, 58]. Changing outcomes of cardiovascular diseases require deeper investigation, including risk factors and the complications of COVID-19 [59]. Although many normal hospital activities have resumed after 2 years of the COVID-19 outbreak, many diseases have become more deadly. Healthcare disruption in the first year could have serious consequences, as repeatedly warned.

As a result of our study, non-COVID-19 mortality peaks correspond to COVID-19 hospital admissions, confirmed COVID-19 cases, and excess mortality caused by COVID-19 in Iran. There are three possible explanations for this phenomenon. Firstly, in this study, cause-specific outcomes were analyzed based on the primary diagnosis. The observed increase in non-COVID-19 in-hospital mortality throughout the entire study could be attributed to COVID-19 infection acting as a confounding factor. In fact, COVID-19 may have been listed as a secondary diagnosis in medical records. Secondly, the indirect effects of the COVID-19 pandemic as a crisis, particularly during peak periods, could have impacted the mortality rates of other diseases. We discussed this phenomenon previously. Additionally, a third reason could be ascribed to the underdiagnosis or underreporting of COVID-19 cases, as previously discussed in studies analyzing excess mortality [60–63]. This discrepancy could lead to an increase in non-COVID-19 mortality cases that are, in fact, related to COVID-19.

Several studies have highlighted Iran’s potential underreporting of COVID-19 cases and deaths due to a shortage of test kits [61–63]. Iran ranks 12th globally in terms of excess deaths, with a P score of 28.9, recorded between January 2020 and September 2021 [64]. Moreover, the number of additional deaths reported in Iran was 1.76 times higher than the number of deaths associated with COVID-19 [64]. These excess deaths align closely with our study findings, which indicate a higher mortality rate in hospitals that may be associated with the COVID-19 pandemic.

It is crucial to acknowledge that this study focuses on in-hospital mortality as defined by primary diagnosis and discharge status. There may be variations between the cause of death reported in this study and the official death certification process in Iran. Additionally, the limited availability of COVID-19 test kits in Iran does not have a significant impact on the results of this study. This is because we excluded from our dataset both laboratory-confirmed and clinically diagnosed COVID-19 cases, which serve as the primary reasons for hospitalization. Furthermore, we found inpatient departments in Iran to be less affected by the shortage of COVID-19 tests compared to outpatient facilities.

It is important to note that not all hospital outcomes can be attributed to the COVID-19 pandemic. During the 2 years of the study, many environmental, economic, social, and political changes occurred. These dynamic changes have always had an impact on disease incidence and outcomes, the demand for services, and the way health services are provided, all of which require further investigation. For this reason, it is better to interpret the results of this study comprehensively and consider the instability of many conditions over time.

This study’s strength is the comprehensive examination of all disease groups. Large-scale hospitalization and mortality data can be used for comparisons and more detailed future analyses. Ultimately, these studies are expected to lead to improved policies and increased public health.

The first limitation of this study is that it relies solely on the primary diagnosis in hospital records to categorize cause-specific hospital admissions and in-hospital mortality. Consequently, patients who contract SARS-CoV-2 infection while being hospitalized for another disease may be classified as non-COVID-19 cases due to this categorization approach. A second limitation is the short period of time before the pandemic. Due to the Rose database’s installation five months before the COVID-19 outbreak, this study lacks data covering a longer period before the pandemic. Different timeframes have been considered to address this limitation.

The research is further constrained by the fact that IHIO only has contracts with 956 of Iran’s 1085 hospitals. Approximately 12% of the country’s hospitals, mainly private and costly establishments, do not have a contract with IHIO. Consequently, when IHIO members are admitted to these hospitals, they have to bear the expenses themselves or rely on secondary insurance coverage. As a result, these hospital admissions are not captured in the Rose database, and this study does not encompass an examination of such cases.

It is also worth noting that the exact date of the initial COVID-19 outbreak in Iran may differ from its official announcement date of the outbreak, and this study did not investigate this discrepancy [61]. It is plausible that individuals seeking treatment during that period may have had COVID-19 but were either misdiagnosed or had empty codes assigned because of the timing of hospital admissions prior to widespread awareness of this disease and the coding protocols conveyed to hospital staff. Furthermore, it is highly likely that some cases were classified as influenza in hospitals because of the concurrent influenza wave preceding the onset of the COVID-19 pandemic.

## Conclusion

In the event of epidemics or pandemics, continuity of health services is imperative. A great lesson to learn from the COVID-19 pandemic is the importance of being prepared for critical situations. Perhaps if countries had provided services to COVID-19 patients in a more organized way from the beginning, the suspension of services would not have had such long-term effects on other diseases. Even though COVID-19 is no longer a global emergency, its direct and indirect effects on people’s health should be considered in the event that similar conditions arise again.

### Supplementary Information

**Additional file 1.**

## Data Availability

All data analyzed during this study are included in this published article and its supplementary information files. The dataset supporting the findings of this study is available from the Iran Health Insurance Organization, but restrictions apply to the availability of these data, which were used under license for the current study, and so are not publicly available. Data are however available from the authors upon reasonable request and with permission of Iran Health Insurance Organization.
