# Supplementary material for: Non-COVID-19 hospitalization and mortality during the COVID-19 pandemic in Iran: a longitudinal assessment of 41 million people in 2019–2022
Source: BMC Public Health. 2024 Feb 5;24:380. doi: 10.1186/s12889-024-17819-0 (PMC10840276; doi:10.1186/s12889-024-17819-0)
Supplement: Supplementary file 1 — Additional file 1. [file 12889_2024_17819_MOESM1_ESM.pdf]

Supplementary materials

Non-COVID-19 Hospitalization and Mortality during the COVID-19 Pandemic in Iran: A Longitudinal Assessment of 41 Million People in 2019–2022

Mahya Razimoghadam, Mehdi Yaseri, Mehdi Rezaee, Aliakbar Fazaeli, Rajabali Daroudi*

Outline

Contents	Page Number
Figure S1. Study population flowchart	3
Table S1. Study diagnosis codes based on ICD 10th	4
Table S2. The Number (percentage) of monthly hospital admissions for non-COVID-19 diseases during each period, by age and sex	5
Table S3. The Number (percentage) of monthly hospital admissions for non-COVID-19 diseases during each period, by type of disease	6
Table S4. The Number (percentage) of monthly in-hospital deaths from non-COVID-19 diseases during each period, by age and sex	7
Table S5. The Number (percentage) of monthly in-hospital deaths from non-COVID-19 diseases during each period, by type of disease	8
Table S6. The hospital admission rate for non-COVID-19 diseases per million/month during each period, by age and sex	9
Table S7. The standardized hospital admission rate for non-COVID-19 diseases per million/month during each period, by type of disease	10
Table S8. The in-hospital mortality rate for non-COVID-19 diseases per 1000/month during each period, by age and sex	11
Table S9. The in-hospital mortality rate for non-COVID-19 diseases per 1000/month during each period, by type of disease	12
Table S10. The Incidence Rate Ratio of non-COVID-19 hospital admissions at the first peak of COVID-19 compared with the pre-pandemic period, by age and sex	13
Table S11. The adjusted Incidence Rate Ratio of non-COVID-19 hospital admissions at the first peak of COVID-19 compared with the pre-pandemic period, by type of disease	14

Table S12. The Odds Ratio of non-COVID-19 in-hospital mortality at the first peak of COVID-19 compared with the pre-pandemic period, by age and sex ———— 15
Table S13. The adjusted Odds Ratio of non-COVID-19 in-hospital mortality at the first peak of COVID-19 compared with the pre-pandemic period, by type of disease ———— 16
Table S14. The Incidence Rate Ratio of non-COVID-19 hospital admissions in the first year of COVID-19 compared with the pre-pandemic period, by age and sex ———— 17
Table S15. The adjusted Incidence Rate Ratio of non-COVID-19 hospital admissions in the first year of COVID-19 compared with the pre-pandemic period, by type of disease ———— 18
Table S16. The Odds Ratio of non-COVID-19 in-hospital mortality in the first year of COVID-19 compared with the pre-pandemic period, by age and sex ———— 19
Table S17. The adjusted Odds Ratio of non-COVID-19 in-hospital mortality in the first year of COVID-19 compared with the pre-pandemic period, by type of disease ———— 20
Table S18. The Incidence Rate Ratio of non-COVID-19 hospital admissions in the second year of COVID-19 compared with the pre-pandemic period, by age and sex ———— 21
Table S19. The adjusted Incidence Rate Ratio of non-COVID-19 hospital admissions in the second year of COVID-19 compared with the pre-pandemic period, by age and sex ———— 22
Table S20. The Odds Ratio of non-COVID-19 in-hospital mortality in the second year of COVID-19 compared with the pre-pandemic period, by age and sex ———— 23
Table S21. The adjusted Odds Ratio of non-COVID-19 in-hospital mortality in the second year of COVID-19 compared with the pre-pandemic period, by type of disease ———— 24
Table S22. The Incidence Rate Ratio of non-COVID-19 hospital admissions in the second year of COVID-19 compared with the first year, by age and sex ———— 25
Table S23. The adjusted Incidence Rate Ratio of non-COVID-19 hospital admissions in the second year of COVID-19 compared with the first year, by type of disease ———— 26
Table S24. The Odds Ratio of non-COVID-19 in-hospital mortality in the second year of COVID-19 compared with the first year, by age and sex ———— 27
Table S25. The adjusted Odds Ratio of non-COVID-19 in-hospital mortality in the second year of COVID-19 compared with the first year, by type of disease ———— 28

Figure S1. Study population flowchart for IHIO members from September 23, 2019 to February 19, 2022

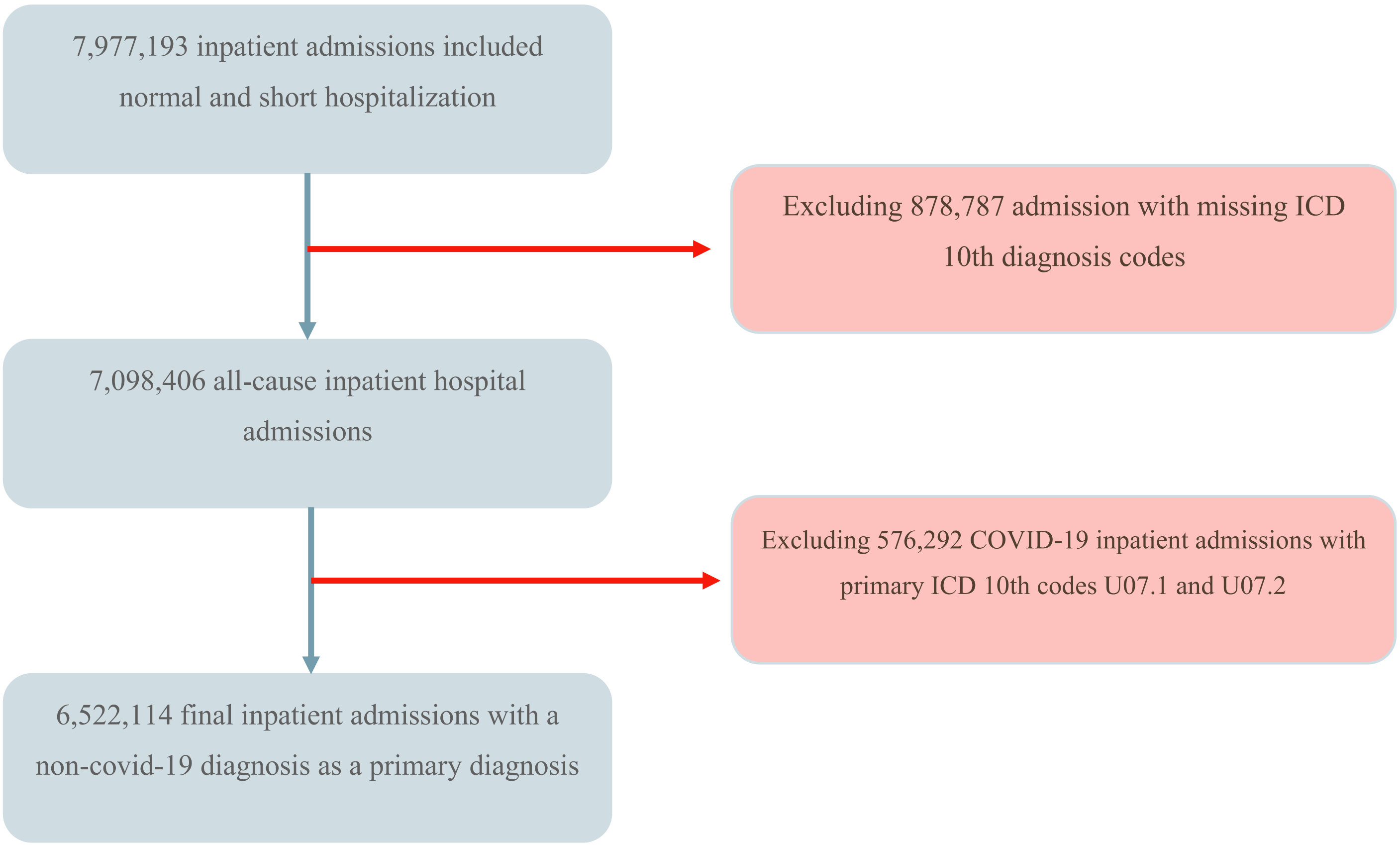

Table S1. Study diagnosis codes based on ICD 10th.

**ICD 10th chapters**	**Short form of chapters name**	**ICD 10th codes**
Infectious and parasitic diseases	Infectious	A00-B99
Neoplasms	Neoplasms	C00-D48
Diseases of the blood and blood-forming organs	Blood	D50-D89
Endocrine, nutritional and metabolic diseases	Metabolic	E00-E90
Mental and behavioral disorders	Mental	F00-F99
Diseases of the nervous system	Nervous Syst.	G00-G99
Diseases of the eye and adnexa	Eye	H00-H59
Diseases of the ear and mastoid process	Ear	H60-H95
Diseases of the circulatory system	Circulatory	I00-I99
Diseases of the respiratory system	Respiratory	J00-J99
Diseases of the digestive system	Digestive	K00-K93
Diseases of the skin and subcutaneous tissue	Skin	L00-L99
Diseases of the musculoskeletal system and connective tissue	Musculoskeletal	M00-M99
Diseases of the genitourinary system	Genitourinary	N00-N99
Pregnancy, childbirth and the puerperium	Pregnancy	O00-O99
Certain conditions originating in the perinatal period	Perinatal	P00-P96
Congenital malformations, deformations and chromosomal abnormalities	Malformations	Q00-Q99
Symptoms, signs and abnormal clinical and laboratory findings	Abnormal symptoms	R00-R99
Injury, poisoning and certain other consequences of external causes	Injury & poisoning	S00-T98
External causes of morbidity and mortality	External causes	V01-Y98
Factors influencing health status and contact with health services	Health services contact	Z00–Z99
**Selected Diseases**		
Pneumonia		J12-J18
Influenza		J09-J11
COPD		J44
Myocardial infarction		I21, I22
Heart failure		I50
Stroke		163, I64
**Excluded disease**		
COVID-19		U07.1, U07.2

Table S2. The Number (percentage) of monthly hospital admissions for non-COVID-19 diseases during each period, by age and sex

**Monthly hospital admission Number (%)**	Pre-pandemic		Post-pandemic							
	Number	%	Total		First Peak		First year		Second year	
			Number	%	Number	%	Number	%	Number	%
Total	200,008	100	230,086	100	99,588	100	184,779	100	275,394	100
**Age groups**										
< 5	22,967	11.5	18,798	8.2	8,898	8.9	14537	7.9	23,058	8.5
5_14	17,703	8.9	12,278	5.3	5,297	5.3	10013	5.4	14,542	5.3
15-44	64,763	32.4	90,521	39.3	50,977	51.2	77062	41.7	103,981	37.6
45-64	43,671	21.8	55,221	24.0	16,351	16.4	41771	22.6	68,671	25.0
≥ 65	50,904	25.5	53,269	23.2	18,065	18.1	41396	22.4	65,141	23.7
**Sex**										
Female	106,561	53.3	127,752	55.5	61,210	61.5	104150	56.4	151,354	54.8
Male	93,447	46.7	102,335	45.0	38,378	38.5	80630	43.6	124,040	45.2

Table S3. The Number (percentage) of monthly hospital admissions for non-COVID-19 diseases during each period, by type of disease

**Monthly hospital admission Number (%)**	Pre-pandemic		Post-pandemic							
	Number	%	Total		First Peak		First year		Second year	
			Number	%	Number	%	Number	%	Number	%
Total	200,008	100	230,086	100	99,588	100	184,779	100	275,394	100
**ICD chapters**										
Infectious	8,391	4.2	6,109	2.7	1,779	1.8	4,444	2.4	7,774	2.8
Neoplasms	5,979	3.0	8,128	3.5	2,426	2.4	6,387	3.5	9,868	3.6
Blood	1,937	1.0	5,991	2.6	1,435	1.4	3,984	2.2	7,998	2.9
Metabolic	4,229	2.1	3,999	1.7	1,376	1.4	3,255	1.8	4,743	1.7
Mental	5,260	2.6	5,143	2.2	1,687	1.7	4,469	2.4	5,817	2.1
Nervous Syst.	3,525	1.8	2,928	1.3	958	1.0	2,411	1.3	3,446	1.3
Eye	7,439	3.7	15,138	6.6	443	0.4	9,486	5.1	20,791	7.5
Ear	927	0.5	493	0.2	88	0.1	399	0.2	587	0.2
Circulatory	23,949	12.0	22,424	9.7	8,244	8.3	18,497	10.0	26,352	9.6
Respiratory	25,961	13.0	7,623	3.3	3,962	4.0	6,215	3.4	9,032	3.3
Digestive	12,624	6.3	14,958	6.5	4,701	4.7	12,084	6.5	17,832	6.5
Skin	2,227	1.1	2,179	0.9	535	0.5	1,750	0.9	2,608	0.9
Musculoskeletal	3,719	1.9	4,708	2.0	588	0.6	3,433	1.9	5,984	2.2
Genitourinary	14,375	7.2	21,605	9.4	6,504	6.5	15,746	8.5	27,464	10.0
Pregnancy	14,003	7.0	24,539	10.7	17,766	17.8	21,009	11.4	28,069	10.2
Perinatal	7,293	3.7	7,348	3.2	5,171	5.2	6,579	3.6	8,117	2.9
Malformations	1,426	0.7	1,508	0.7	353	0.4	1,249	0.7	1,766	0.6
Abnormal symptoms	17,213	8.6	17,390	7.6	6,365	6.4	12,602	6.8	22,179	8.1
Injury & poisoning	15,712	7.9	19,379	8.4	10,644	10.7	16,722	9.0	22,036	8.0
External causes	1,844	0.9	2,488	1.1	1,796	1.8	2,253	1.2	2,724	1.0
Health services contact	21,975	11.0	36,007	15.6	22,767	22.9	31,807	17.2	40,207	14.6
**Selected diseases**										
Pneumonia	10,987	5.5	2,517	1.1	1,872	1.9	2,069	1.1	2,966	1.1
Influenza	3,607	1.8	150	0.1	146	0.1	215	0.1	86	0.0
COPD	3,740	1.9	1,318	0.6	640	0.6	1,069	0.6	1,567	0.6
Myocardial infarction	1,648	0.8	1,635	0.7	1,064	1.1	1,482	0.8	1,789	0.6
Heart failure	914	0.5	718	0.3	351	0.4	639	0.3	798	0.3
Stroke	1,824	0.9	1,606	0.7	922	0.9	1,390	0.8	1,822	0.7

Table S4. The Number (percentage) of monthly in-hospital deaths from non-COVID-19 diseases during each period, by age and sex

**Monthly Death Number (%)**	Pre-pandemic		Post-pandemic							
			Total		First peak		First year		Second year	
	Number	%	Number	%	Number	%	Number	%	Number	%
Total	4,041	100	4,978	100	3,186	100	4,274	100	5,683	100
**Age groups**										
< 5	313	7.7	354	7.1	326	10.2	338	7.9	370	6.6
5_14	61	1.5	55	1.1	53	1.7	51	1.2	59	1.0
15-44	368	9.1	487	9.8	365	11.5	410	9.6	563	9.9
45-64	814	20.1	1,155	23.2	689	21.6	943	22.1	1,367	23.8
≥ 65	2,486	61.5	2,928	58.8	1,753	55.0	2,531	59.2	3,324	58.7
**Sex**										
Female	1,797	44.5	2,212	44.4	1,385	43.5	1,874	43.8	2,550	44.8
Male	2,244	55.5	2,766	55.6	1,801	56.5	2,400	56.2	3,133	55.2

Table S5. The Number (percentage) of monthly in-hospital deaths from non-COVID-19 diseases during each period, by type of disease

**Monthly Death Number (%)**	Pre-pandemic		Post-pandemic							
	Number	%	Total		First Peak		First year		Second year	
			Number	%	Number	%	Number	%	Number	%
Total	4,041	100	5,043	100	3,186	100	4,274	100	5,683	100
**ICD chapters**										
Infectious	316	7.8	269	5.4	144	4.5	210	4.9	327	5.8
Neoplasms	296	7.3	348	7.0	158	5.0	285	6.7	411	7.2
Blood	41	1.0	51	1.0	40	1.3	43	1.0	59	1.0
Metabolic	104	2.6	185	3.7	98	3.1	153	3.6	216	3.8
Mental	14	0.3	26	0.5	13	0.4	22	0.5	30	0.5
Nervous Syst.	77	1.9	89	1.8	34	1.1	76	1.8	102	1.8
Eye	1	0.0	2	0.0	0	0.0	1	0.0	2	0.0
Ear	0	0.0	1	0.0	0	0.0	0	0.0	1	0.0
Circulatory	1,113	27.5	1,341	26.9	930	29.2	1,154	27.0	1,529	26.9
Respiratory	559	13.8	533	10.7	388	12.2	452	10.6	615	11.0
Digestive	217	5.4	253	5.1	134	4.2	216	5.1	289	5.1
Skin	25	0.6	22	0.5	13	0.4	18	0.4	27	0.5
Musculoskeletal	22	0.5	45	0.9	11	0.4	35	0.8	54	0.9
Genitourinary	185	4.6	212	4.3	131	4.1	181	4.2	243	4.3
Pregnancy	3	0.1	5	0.1	3	0.1	5	0.1	6	0.1
Perinatal	165	4.1	210	4.2	201	6.3	203	4.8	217	3.8
Malformations	52	1.3	54	1.1	40	1.3	50	1.2	58	1.0
Abnormal symptoms	495	12.2	780	15.7	372	11.7	635	14.8	926	16.3
Injury & poisoning	191	4.7	254	5.1	202	6.3	228	5.3	281	5.0
External causes	42	1.0	30	0.6	26	0.8	32	0.7	29	0.4
Health services contact	124	3.1	268	5.4	248	7.8	276	6.5	260	4.5
**Selected diseases**										
Pneumonia	204	5.0	176	3.6	175	5.5	156	3.6	197	3.4
Influenza	42	1.0	4	0.1	4	0.1	6	0.1	1	0.0
COPD	92	2.3	58	1.2	37	1.2	49	1.1	67	1.2
Myocardial infarction	87	2.2	98	2.0	66	2.1	84	2.0	112	1.9
Heart failure	40	1.0	47	1.0	28	0.9	42	1.0	53	0.9
Stroke	156	3.9	166	3.4	117	3.7	139	3.3	194	3.3

Table S6. The hospital admission rate for non-COVID-19 diseases per one million person-month during each period, by age and sex

**Admission rate per one million-month**		Pre-pandemic	Post-pandemic			
			Total	First peak	First year	Second year
Female	< 5	6884.82	6435.23	3176.95	4980.98	7889.48
	5_14	2260.24	1478.72	641.50	1193.78	1763.66
	15-44	4239.55	6206.66	4001.45	5438.39	6974.93
	45-64	5618.92	6767.21	1806.52	5026.23	8508.19
	≥ 65	16148.99	15316.89	4879.94	11712.73	18921.05
Male	< 5	8621.08	8057.61	3725.42	6150.67	9964.56
	5_14	3049.13	2098.98	900.82	1727.25	2470.71
	15-44	2075.70	2671.93	1047.52	2133.92	3209.94
	45-64	5588.32	6963.87	2263.71	5378.18	8549.55
	≥ 65	16665.19	17117.32	6119.64	13573.13	20661.51

Table S7. The standardized hospital admission rate for non-COVID-19 diseases per million person-month during each period, by type of disease

**Admission rate per million person -month**	Pre-pandemic	Post -pandemic			
		Total	First peak	First year	Second year
Total	7115.19	7311.44	2856.35	5731.53	8891.36
**ICD chapters**					
Infectious	398.22	313.11	83.04	218.30	407.91
Neoplasms	218.93	274.87	82.23	216.91	332.84
Blood	66.27	143.19	48.89	99.39	186.99
Metabolic	167.20	148.06	53.18	122.27	173.85
Mental	93.84	86.60	27.81	74.66	98.53
Nervous Syst.	115.34	92.29	34.93	76.04	108.53
Eye	352.97	671.30	15.88	414.26	928.35
Ear	35.63	15.81	3.51	12.88	18.73
Circulatory	1085.11	912.47	354.92	764.30	1060.64
Respiratory	1138.24	329.37	167.92	257.78	400.96
Digestive	413.47	448.40	133.84	357.28	539.51
Skin	59.10	49.85	13.58	40.51	59.19
Musculoskeletal	112.73	139.79	16.15	98.88	180.71
Genitourinary	487.43	692.16	227.35	510.44	873.87
Pregnancy	155.69	263.54	185.68	227.76	299.01
Perinatal	497.20	560.33	440.31	501.18	618.98
Malformations	63.61	79.29	25.61	66.96	91.52
Abnormal symptoms	664.82	649.69	238.72	470.72	828.67
Injury & poisoning	424.50	514.57	264.58	440.99	588.14
External causes	52.34	70.23	44.39	63.26	77.21
Health services contact	528.71	864.69	440.45	707.19	1022.20
**Selected diseases**					
Pneumonia	527.62	126.68	79.25	97.42	155.96
Influenza	112.56	6.18	5.41	7.29	4.84
COPD	185.64	60.89	29.76	49.67	72.20
Myocardial infarction	104.01	98.00	61.60	91.92	103.64
Heart failure	52.64	42.30	24.99	37.34	47.41
Stroke	129.92	96.64	75.52	87.26	105.54

Table S8. The in-hospital mortality rate for non-COVID-19 diseases per 1000 admission-month during each period, by age and sex

**Mortality rate per 1000 admission-month**		Pre-pandemic	Post pandemic			
			Total	First peak	First year	Second year
Female	< 5	14.07	19.99	40.27	24.31	17.23
	5_14	3.59	5.04	13.27	5.64	4.64
	15-44	3.78	3.06	3.06	2.90	3.19
	45-64	13.64	17.18	36.55	18.64	16.32
	≥ 65	46.23	52.64	97.69	58.83	48.83
Male	< 5	13.28	17.99	33.84	22.42	15.22
	5_14	3.33	4.13	7.84	4.81	3.66
	15-44	9.22	10.23	21.32	10.91	9.78
	45-64	23.84	24.68	46.77	26.39	23.60
	≥ 65	51.42	57.17	96.48	63.28	53.17

Table S9. The in-hospital mortality rate for non-COVID-19 diseases per 1000 admission-month during each period, by type of disease

**Mortality rate per 1000 admission-month**	Pre-pandemic	Post -pandemic			
		Total	First peak	First year	Second year
Total	20.20	21.64	31.99	23.13	20.63
**ICD chapters**					
Infectious	37.64	43.99	80.94	47.27	42.11
Neoplasms	49.51	42.83	65.13	44.63	41.66
Blood	21.17	8.49	27.87	10.79	7.34
Metabolic	24.54	46.17	71.22	47.11	45.52
Mental	2.62	5.07	7.71	4.96	5.16
Nervous Syst.	21.73	30.38	35.49	31.55	29.56
Eye	0.11	0.10	0.00	0.10	0.10
Ear	0.43	1.01	0.00	0.84	1.14
Circulatory	46.47	59.82	112.81	62.38	58.03
Respiratory	21.54	69.96	97.93	72.68	68.09
Digestive	17.16	16.89	28.50	17.86	16.23
Skin	11.32	10.31	24.30	10.15	10.42
Musculoskeletal	5.86	9.50	18.71	10.32	9.02
Genitourinary	12.86	9.81	20.14	11.48	8.85
Pregnancy	0.20	0.21	0.17	0.21	0.21
Perinatal	22.65	28.58	38.87	30.87	26.73
Malformations	36.32	35.77	113.31	39.71	32.98
Abnormal symptoms	28.73	44.87	58.44	50.35	41.75
Injury & poisoning	12.18	13.12	18.98	13.60	12.76
External causes	22.89	12.12	14.48	14.05	10.53
Health services contact	5.66	7.45	10.89	8.69	6.47
**Selected diseases**					
Pneumonia	18.53	70.03	93.48	75.25	66.40
Influenza	11.59	24.92	27.40	28.68	15.52
COPD	24.71	44.24	57.81	46.17	42.92
Myocardial infarction	52.56	59.99	62.03	56.70	62.71
Heart failure	43.75	65.74	79.77	65.52	65.91
Stroke	85.54	103.44	126.90	99.66	106.32

Table S10. The Incidence Rate Ratio of non-COVID-19 hospital admissions at the first peak of COVID-19 compared with the pre-pandemic period, by age and sex

Sex	Age group	Incidence Rate Ratio	95% confidence interval		P value	Adjusted P value
			Lower bound	Upper bound		
Female	< 5	0.46	0.26	0.81	0.01	0.02
	5_14	0.28	0.16	0.49	0.00	0.00
	15-44	0.94	0.55	1.62	0.84	0.87
	45-64	0.32	0.21	0.48	0.00	0.00
	≥ 65	0.30	0.22	0.42	0.00	0.00
Male	< 5	0.43	0.25	0.74	0.00	0.00
	5_14	0.30	0.19	0.46	0.00	0.00
	15-44	0.50	0.35	0.72	0.00	0.00
	45-64	0.41	0.29	0.57	0.00	0.00
	≥ 65	0.37	0.26	0.51	0.00	0.00

Table S11. The adjusted Incidence Rate Ratio of non-COVID-19 hospital admissions at the first peak of COVID-19 compared with the pre-pandemic period, by type of disease

	Incidence Rate Ratio	95% confidence interval		P value	Adjusted P value
		Lower bound	Upper bound		
Total non-COVID_19	0.40	0.25	0.64	0.00	0.00
**ICD chapters**					
Infectious	0.21	0.11	0.40	0.00	0.00
Neoplasms	0.38	0.18	0.78	0.01	0.02
Blood	0.74	0.49	1.11	0.15	0.19
Metabolic	0.32	0.16	0.62	0.00	0.00
Mental	0.30	0.12	0.72	0.01	0.02
Nervous Syst.	0.30	0.19	0.47	0.00	0.00
Eye	0.04	0.02	0.11	0.00	0.00
Ear	0.09	0.05	0.16	0.00	0.00
Circulatory	0.33	0.11	0.98	0.05	0.07
Respiratory	0.15	0.08	0.28	0.00	0.00
Digestive	0.32	0.20	0.54	0.00	0.00
Skin	0.23	0.15	0.35	0.00	0.00
Musculoskeletal	0.14	0.08	0.26	0.00	0.00
Genitourinary	0.47	0.24	0.89	0.02	0.03
Pregnancy	1.19	0.29	4.85	0.81	0.84
Perinatal	0.89	0.17	4.60	0.89	0.91
Malformations	0.40	0.15	1.06	0.07	0.09
Abnormal symptoms	0.36	0.22	0.58	0.00	0.00
Injury & poisoning	0.62	0.44	0.89	0.01	0.02
External causes	0.85	0.58	1.24	0.41	0.48
Health services contact	0.83	0.49	1.42	0.51	0.58
**Selected diseases**					
Pneumonia	0.15	0.07	0.30	0.00	0.00
Influenza	0.05	0.02	0.10	0.00	0.00
COPD	0.16	0.06	0.46	0.00	0.00
Myocardial infarction	0.59	0.16	2.17	0.43	0.50
Heart failure	0.47	0.12	1.90	0.29	0.35
Stroke	0.58	0.14	2.41	0.46	0.53

Table S12. The Odds Ratio of non-COVID-19 in-hospital mortality at the first peak of COVID-19 compared with the pre-pandemic period, by age and sex

Sex	Age group	Odds Ratio	95% confidence interval		P value	Adjusted P value
			Lower bound	Upper bound		
Female	< 5	2.94	2.46	3.51	0.00	0.00
	5_14	3.73	2.48	5.63	0.00	0.00
	15-44	0.81	0.67	0.98	0.03	0.04
	45-64	2.74	2.41	3.13	0.00	0.00
	≥ 65	2.23	2.07	2.41	0.00	0.00
Male	< 5	2.60	2.20	3.07	0.00	0.00
	5_14	2.37	1.55	3.61	0.00	0.00
	15-44	2.34	2.03	2.69	0.00	0.00
	45-64	2.01	1.81	2.23	0.00	0.00
	≥ 65	1.97	1.83	2.12	0.00	0.00

Table S13. The adjusted Odds Ratio of non-COVID-19 in-hospital mortality at the first peak of COVID-19 compared with the pre-pandemic period, by type of disease

	Odds Ratio	95% confidence interval		P value	Adjusted P value
		Lower bound	Upper bound		
Total non-COVID_19	2.05	1.97	2.13	0.00	0.00
**ICD chapters**					
Infectious	1.91	1.58	2.30	0.00	0.00
Neoplasms	1.45	1.22	1.72	0.00	0.00
Blood	1.58	1.12	2.25	0.01	0.02
Metabolic	3.10	2.47	3.88	0.00	0.00
Mental	3.18	1.74	5.82	0.00	0.00
Nervous Syst.	1.56	1.09	2.23	0.02	0.03
Circulatory	2.62	2.43	2.82	0.00	0.00
Respiratory	3.28	2.93	3.68	0.00	0.00
Digestive	1.96	1.62	2.35	0.00	0.00
Skin	2.51	1.39	4.56	0.00	0.00
Musculoskeletal	3.74	1.99	7.03	0.00	0.00
Genitourinary	1.39	1.16	1.68	0.00	0.00
Pregnancy	1.06	0.30	3.75	0.93	0.95
Perinatal	1.74	1.49	2.04	0.00	0.00
Malformations	2.21	1.54	3.16	0.00	0.00
Abnormal symptoms	1.93	1.72	2.16	0.00	0.00
Injury & poisoning	1.73	1.49	2.02	0.00	0.00
External causes	0.65	0.43	0.98	0.04	0.06
Health services contact	2.62	2.25	3.04	0.00	0.00
**Selected diseases**					
Pneumonia	3.36	2.83	4.00	0.00	0.00
Influenza	1.36	0.49	3.73	0.56	0.63
COPD	2.42	1.71	3.42	0.00	0.00
Myocardial infarction	1.31	1.00	1.71	0.05	0.07
Heart failure	1.90	1.26	2.87	0.00	0.00
Stroke	1.59	1.29	1.96	0.00	0.00

Table S14. The Incidence Rate Ratio of non-COVID-19 hospital admissions in the first year of COVID-19 compared with the pre-pandemic period, by age and sex

Sex	Age group	Incidence Rate Ratio	95% confidence interval		P value	Adjusted P value
			Lower bound	Upper bound		
Female	< 5	0.72	0.58	0.90	0.00	0.00
	5_14	0.53	0.42	0.67	0.00	0.00
	15-44	1.28	1.06	1.55	0.01	0.02
	45-64	0.89	0.65	1.24	0.50	0.57
	≥ 65	0.73	0.55	0.96	0.02	0.03
Male	< 5	0.71	0.57	0.89	0.00	0.00
	5_14	0.57	0.46	0.70	0.00	0.00
	15-44	1.03	0.81	1.31	0.82	0.85
	45-64	0.96	0.73	1.27	0.79	0.83
	≥ 65	0.81	0.63	1.05	0.11	0.14

Table S15. The adjusted Incidence Rate Ratio of non-COVID-19 hospital admissions in the first year of COVID-19 compared with the pre-pandemic period, by type of disease

	Incidence Rate Ratio	95% confidence interval		P value	Adjusted P value
		Lower bound	Upper bound		
Total non-COVID_19	0.80	0.73	0.89	0.00	0.00
**ICD chapters**					
Infectious	0.55	0.40	0.76	0.00	0.00
Neoplasms	0.99	0.70	1.40	0.96	0.97
Blood	1.50	1.24	1.83	0.00	0.00
Metabolic	0.73	0.53	1.01	0.06	0.08
Mental	0.80	0.52	1.22	0.30	0.36
Nervous Syst.	0.66	0.53	0.81	0.00	0.00
Eye	1.17	0.72	1.91	0.52	0.59
Ear	0.36	0.28	0.46	0.00	0.00
Circulatory	0.70	0.42	1.19	0.19	0.24
Respiratory	0.23	0.17	0.31	0.00	0.00
Digestive	0.86	0.68	1.10	0.23	0.29
Skin	0.69	0.56	0.84	0.00	0.00
Musculoskeletal	0.88	0.64	1.21	0.43	0.50
Genitourinary	1.05	0.75	1.45	0.78	0.83
Pregnancy	1.46	0.73	2.93	0.28	0.34
Perinatal	1.01	0.46	2.21	0.98	0.98
Malformations	1.05	0.66	1.68	0.83	0.86
Abnormal symptoms	0.71	0.55	0.91	0.01	0.02
Injury & poisoning	1.04	0.89	1.22	0.63	0.69
External causes	1.21	1.00	1.46	0.05	0.07
Health services contact	1.34	1.02	1.76	0.04	0.06
**Selected diseases**					
Pneumonia	0.18	0.13	0.26	0.00	0.00
Influenza	0.06	0.04	0.10	0.00	0.00
COPD	0.27	0.16	0.46	0.00	0.00
Myocardial infarction	0.88	0.49	1.60	0.68	0.73
Heart failure	0.71	0.40	1.27	0.25	0.31
Stroke	0.67	0.35	1.27	0.22	0.27

Table S16. The Odds Ratio of non-COVID-19 in-hospital mortality in the first year of COVID-19 compared with the pre-pandemic period, by age and sex

Sex	Age group	Odds Ratio	95% confidence interval		P value	Adjusted P value
			Lower bound	Upper bound		
Female	< 5	1.75	1.60	1.91	0.00	0.00
	5_14	1.57	1.28	1.94	0.00	0.00
	15-44	0.77	0.71	0.83	0.00	0.00
	45-64	1.37	1.30	1.46	0.00	0.00
	≥ 65	1.29	1.25	1.33	0.00	0.00
Male	< 5	1.70	1.58	1.84	0.00	0.00
	5_14	1.45	1.21	1.74	0.00	0.00
	15-44	1.19	1.10	1.27	0.00	0.00
	45-64	1.11	1.06	1.16	0.00	0.00
	≥ 65	1.25	1.21	1.28	0.00	0.00

Table S17. The adjusted Odds Ratio of non-COVID-19 in-hospital mortality in the first year of COVID-19 compared with the pre-pandemic period, by type of disease

	Odds Ratio	95% confidence interval		P value	Adjusted P value
		Lower bound	Upper bound		
Total non-COVID_19	1.26	1.23	1.28	0.00	0.00
**ICD chapters**					
Infectious	1.01	0.95	1.08	0.71	0.76
Neoplasms	0.93	0.87	0.99	0.02	0.03
Blood	0.80	0.68	0.95	0.01	0.02
Metabolic	2.03	1.83	2.24	0.00	0.00
Mental	2.05	1.57	2.68	0.00	0.00
Nervous Syst.	1.52	1.34	1.71	0.00	0.00
Circulatory	1.42	1.38	1.47	0.00	0.00
Respiratory	2.81	2.68	2.95	0.00	0.00
Digestive	1.16	1.08	1.24	0.00	0.00
Skin	1.17	0.93	1.47	0.17	0.22
Musculoskeletal	1.78	1.44	2.20	0.00	0.00
Genitourinary	0.89	0.82	0.96	0.00	0.00
Pregnancy	1.15	0.63	2.07	0.65	0.70
Perinatal	1.37	1.27	1.49	0.00	0.00
Malformations	0.95	0.82	1.11	0.55	0.62
Abnormal symptoms	1.57	1.50	1.64	0.00	0.00
Injury & poisoning	1.18	1.10	1.27	0.00	0.00
External causes	0.62	0.52	0.74	0.00	0.00
Health services contact	1.63	1.50	1.78	0.00	0.00
**Selected diseases**					
Pneumonia	3.10	2.86	3.36	0.00	0.00
Influenza	1.85	1.41	2.43	0.00	0.00
COPD	1.94	1.71	2.20	0.00	0.00
Myocardial infarction	1.16	1.03	1.30	0.02	0.03
Heart failure	1.55	1.31	1.84	0.00	0.00
Stroke	1.22	1.12	1.34	0.00	0.00

Table S18. The Incidence Rate Ratio of non-COVID-19 hospital admissions in the second year of COVID-19 compared with the pre-pandemic period, by age and sex

Sex	Age group	Incidence Rate Ratio	95% confidence interval		P value	Adjusted P value
			Lower bound	Upper bound		
Female	< 5	1.15	0.83	1.57	0.40	0.47
	5_14	0.78	0.60	1.01	0.06	0.08
	15-44	1.65	1.22	2.22	0.00	0.00
	45-64	1.51	1.16	1.97	0.00	0.00
	≥ 65	1.17	0.86	1.60	0.32	0.38
Male	< 5	1.16	0.84	1.59	0.37	0.44
	5_14	0.80	0.61	1.06	0.12	0.15
	15-44	1.55	1.20	2.00	0.00	0.00
	45-64	1.52	1.13	2.03	0.01	0.02
	≥ 65	1.24	0.90	1.70	0.18	0.23

Table S19. The adjusted Incidence Rate Ratio of non-COVID-19 hospital admissions in the second year of COVID-19 compared with the pre-pandemic period, by type of disease

	Incidence Rate Ratio	95% confidence interval		P value	Adjusted P value
		Lower bound	Upper bound		
Total non-COVID_19	1.25	1.13	1.40	0.00	0.00
**ICD chapters**					
Infectious	1.02	0.73	1.44	0.89	0.91
Neoplasms	1.52	1.09	2.13	0.01	0.02
Blood	2.84	2.42	3.33	0.00	0.00
Metabolic	1.04	0.76	1.43	0.80	0.84
Mental	1.05	0.70	1.57	0.81	0.84
Nervous Syst.	0.94	0.76	1.16	0.57	0.64
Eye	2.63	1.62	4.27	0.00	0.00
Ear	0.52	0.41	0.66	0.00	0.00
Circulatory	0.98	0.58	1.64	0.93	0.95
Respiratory	0.35	0.26	0.47	0.00	0.00
Digestive	1.30	1.04	1.64	0.02	0.03
Skin	1.00	0.83	1.21	0.97	0.98
Musculoskeletal	1.60	1.17	2.20	0.00	0.00
Genitourinary	1.79	1.30	2.49	0.00	0.00
Pregnancy	1.92	0.96	3.85	0.07	0.09
Perinatal	1.24	0.58	2.69	0.58	0.65
Malformations	1.44	0.93	2.23	0.11	0.14
Abnormal symptoms	1.25	0.98	1.58	0.07	0.09
Injury & poisoning	1.39	1.20	1.60	0.00	0.00
External causes	1.48	1.22	1.79	0.00	0.00
Health services contact	1.93	1.50	2.50	0.00	0.00
**Selected diseases**					
Pneumonia	0.30	0.21	0.42	0.00	0.00
Influenza	0.04	0.03	0.06	0.00	0.00
COPD	0.39	0.23	0.65	0.00	0.00
Myocardial infarction	1.00	0.55	1.80	0.99	0.99
Heart failure	0.90	0.50	1.63	0.73	0.78
Stroke	0.81	0.43	1.54	0.52	0.59

Table S20. The Odds Ratio of non-COVID-19 in-hospital mortality in the second year of COVID-19 compared with the pre-pandemic period, by age and sex

Sex	Age group	Odds Ratio	95% confidence interval		P value	Adjusted P value
			Lower bound	Upper bound		
Female	< 5	1.22	1.12	1.34	0.00	0.00
	5_14	1.29	1.06	1.59	0.01	0.02
	15-44	0.84	0.78	0.91	0.00	0.00
	45-64	1.20	1.13	1.27	0.00	0.00
	≥ 65	1.06	1.03	1.10	0.00	0.00
Male	< 5	1.15	1.06	1.24	0.00	0.00
	5_14	1.10	0.92	1.32	0.26	0.32
	15-44	1.06	0.99	1.14	0.09	0.12
	45-64	0.99	0.95	1.03	0.66	0.71
	≥ 65	1.04	1.00	1.07	0.02	0.03

Table S21. The adjusted Odds Ratio of non-COVID-19 in-hospital mortality in the second year of COVID-19 compared with the pre-pandemic period, by type of disease

	Odds Ratio	95% confidence interval		P value	Adjusted P value
		Lower bound	Upper bound		
Total non-COVID_19	1.05	1.04	1.07	0.00	0.00
**ICD chapters**					
Infectious	1.04	0.98	1.10	0.22	0.27
Neoplasms	0.87	0.82	0.93	0.00	0.00
Blood	0.73	0.62	0.86	0.00	0.00
Metabolic	1.99	1.80	2.19	0.00	0.00
Mental	2.15	1.65	2.78	0.00	0.00
Nervous Syst.	1.42	1.26	1.60	0.00	0.00
Circulatory	1.35	1.31	1.39	0.00	0.00
Respiratory	2.95	2.82	3.09	0.00	0.00
Digestive	1.04	0.97	1.12	0.26	0.32
Skin	1.21	0.98	1.49	0.08	0.11
Musculoskeletal	1.48	1.20	1.82	0.00	0.00
Genitourinary	0.69	0.64	0.75	0.00	0.00
Pregnancy	1.17	0.66	2.09	0.59	0.65
Perinatal	1.19	1.10	1.28	0.00	0.00
Malformations	0.82	0.71	0.95	0.01	0.02
Abnormal symptoms	1.37	1.31	1.43	0.00	0.00
Injury & poisoning	1.12	1.04	1.20	0.00	0.00
External causes	0.45	0.38	0.54	0.00	0.00
Health services contact	0.99	0.90	1.08	0.76	0.81
**Selected diseases**					
Pneumonia	3.64	3.37	3.93	0.00	0.00
Influenza	1.42	0.85	2.40	0.18	0.23
COPD	1.82	1.62	2.05	0.00	0.00
Myocardial infarction	1.33	1.19	1.49	0.00	0.00
Heart failure	1.59	1.35	1.87	0.00	0.00
Stroke	1.35	1.24	1.47	0.00	0.00

Table S22. The Incidence Rate Ratio of non-COVID-19 hospital admissions in the second year of COVID-19 compared with the first year, by age and sex

Sex	Age group	Incidence Rate Ratio	95% confidence interval		P value	Adjusted P value
			Lower bound	Upper bound		
Female	< 5	1.58	1.25	2.01	0.00	0.00
	5_14	1.48	1.22	1.79	0.00	0.00
	15-44	1.28	1.01	1.64	0.05	0.07
	45-64	1.69	1.38	2.08	0.00	0.00
	≥ 65	1.62	1.27	2.05	0.00	0.00
Male	< 5	1.62	1.28	2.05	0.00	0.00
	5_14	1.43	1.16	1.78	0.00	0.00
	15-44	1.50	1.23	1.84	0.00	0.00
	45-64	1.58	1.26	1.99	0.00	0.00
	≥ 65	1.51	1.19	1.92	0.00	0.00

Table S23. The adjusted Incidence Rate Ratio of non-COVID-19 hospital admissions in the second year of COVID-19 compared with the first year, by type of disease

	Incidence Rate Ratio	95% confidence interval		P value	Adjusted P value
		Lower bound	Upper bound		
Total non-COVID_19	1.55	1.43	1.67	0.00	0.00
**ICD chapters**					
Infectious	1.87	1.44	2.42	0.00	0.00
Neoplasms	1.53	1.20	1.97	0.00	0.00
Blood	1.88	1.65	2.14	0.00	0.00
Metabolic	1.42	1.12	1.81	0.00	0.00
Mental	1.32	0.96	1.81	0.08	0.11
Nervous Syst.	1.43	1.22	1.67	0.00	0.00
Eye	2.24	1.54	3.26	0.00	0.00
Ear	1.46	1.21	1.76	0.00	0.00
Circulatory	1.39	0.93	2.07	0.12	0.15
Respiratory	1.56	1.23	1.97	0.00	0.00
Digestive	1.51	1.27	1.80	0.00	0.00
Skin	1.46	1.26	1.70	0.00	0.00
Musculoskeletal	1.83	1.43	2.34	0.00	0.00
Genitourinary	1.71	1.32	2.21	0.00	0.00
Pregnancy	1.31	0.77	2.24	0.32	0.38
Perinatal	1.24	0.68	2.24	0.49	0.56
Malformations	1.37	0.96	1.95	0.08	0.11
Abnormal symptoms	1.76	1.45	2.13	0.00	0.00
Injury & poisoning	1.33	1.20	1.48	0.00	0.00
External causes	1.22	1.05	1.41	0.02	0.03
Health services contact	1.44	1.17	1.78	0.00	0.00
**Selected diseases**					
Pneumonia	1.60	1.21	2.12	0.00	0.00
Influenza	0.65	0.44	0.98	0.03	0.04
COPD	1.45	0.97	2.19	0.07	0.09
Myocardial infarction	1.12	0.72	1.76	0.60	0.66
Heart failure	1.27	0.81	2.00	0.30	0.36
Stroke	1.21	0.75	1.95	0.43	0.50

Table S24. The Odds Ratio of non-COVID-19 in-hospital mortality in the second year of COVID-19 compared with the first year, by age and sex

Sex	Age group	Odds Ratio	95% confidence interval		P value	Adjusted P value
			Lower bound	Upper bound		
Female	< 5	0.70	0.66	0.75	0.00	0.00
	5_14	0.82	0.70	0.97	0.04	0.06
	15-44	1.10	1.04	1.17	0.00	0.00
	45-64	0.87	0.84	0.91	0.00	0.00
	≥ 65	0.82	0.80	0.84	0.00	0.00
Male	< 5	0.67	0.64	0.71	0.00	0.00
	5_14	0.76	0.66	0.88	0.00	0.00
	15-44	0.90	0.85	0.94	0.00	0.00
	45-64	0.89	0.86	0.92	0.00	0.00
	≥ 65	0.83	0.81	0.85	0.00	0.00

Table S25. The adjusted Odds Ratio of non-COVID-19 in-hospital mortality in the second year of COVID-19 compared with the first year, by type of disease

	Odds Ratio	95% confidence interval		P value	Adjusted P value
		Lower bound	Upper bound		
Total non-COVID_19	0.85	0.84	0.86	0.00	0.00
**ICD chapters**					
Infectious	1.02	0.97	1.08	0.44	0.51
Neoplasms	0.94	0.90	0.98	0.00	0.00
Blood	0.84	0.75	0.94	0.00	0.00
Metabolic	0.98	0.92	1.05	0.60	0.66
Mental	1.04	0.89	1.22	0.61	0.67
Nervous Syst.	0.93	0.85	1.02	0.11	0.14
Circulatory	0.94	0.92	0.97	0.00	0.00
Respiratory	1.06	1.02	1.10	0.00	0.00
Digestive	0.90	0.85	0.95	0.00	0.00
Skin	1.05	0.88	1.25	0.62	0.68
Musculoskeletal	0.83	0.73	0.94	0.00	0.00
Genitourinary	0.78	0.74	0.83	0.00	0.00
Pregnancy	0.99	0.70	1.42	0.97	0.98
Perinatal	0.86	0.82	0.91	0.00	0.00
Malformations	0.86	0.77	0.96	0.01	0.02
Abnormal symptoms	0.88	0.85	0.91	0.00	0.00
Injury & poisoning	0.94	0.90	0.99	0.02	0.03
External causes	0.72	0.62	0.84	0.00	0.00
Health services contact	0.60	0.57	0.63	0.00	0.00
**Selected diseases**					
Pneumonia	1.18	1.11	1.26	0.00	0.00
Influenza	0.75	0.43	1.33	0.33	0.39
COPD	0.94	0.84	1.05	0.26	0.33
Myocardial infarction	1.15	1.05	1.25	0.00	0.00
Heart failure	1.02	0.90	1.15	0.79	0.83
Stroke	1.10	1.03	1.18	0.00	0.00
